# Preparation and Mechanical Behavior of Ultra-High Strength Low-Carbon Steel

**DOI:** 10.3390/ma13020459

**Published:** 2020-01-18

**Authors:** Zhiqing Lv, Lihua Qian, Shuai Liu, Le Zhan, Siji Qin

**Affiliations:** 1 Key Laboratory of Advanced Forging & Stamping Technology and Science, Ministry of Education of China Yanshan University, Qinhuangdao 066004, China; 18753360971@163.com (L.Q.); liushuai951003@163.com (S.L.); Zhanle223@163.com (L.Z.); plastics@ysu.edu.cn (S.Q.); 2 State Key Laboratory of Metastable Material Science and Technology, Yanshan University, Qinhuangdao 066004, China

**Keywords:** lath martensite, cold deformation, aging, ultra-high strength steel

## Abstract

The low-carbon steel (~0.12 wt%) with complete martensite structure, obtained by quenching, was cold rolled to get the high-strength steel sheets. Then, the mechanical properties of the sheets were measured at different angles to the rolling direction, and the microstructural evolution of low-carbon martensite with cold rolling reduction was observed. The results show that the hardness and the strength gradually increase with increasing rolling reduction, while the elongation and impact toughness obviously decrease. The strength of the sheets with the same rolling reduction are different at the angles of 0°, 45°, and 90° to the rolling direction. The tensile strength (elongation) along the rolling direction is higher than that in the other two directions, but the differences between them are not obvious. When the aging was performed at a low temperature, the strength of the initial martensite and deformed martensite increased with increasing aging time during the early stages of aging, followed by a gradual decrease with further aging. However, the elongation increases with increasing aging time. The change of hardness is consistent with that of strength for the cold-rolled martensite, while the hardness of the initial martensite decreases gradually with increasing aging time.

## 1. Introduction

At present, the production technology of the traditional steel has matured. The research and development of a new generation of steel are the primary objectives of steel manufacturers in the 21st century. In recent years, numerous advanced technologies for preparing ultra-high strength steel have emerged. As is well known, grain refinement can significantly improve strength of the materials. Therefore, the preparation of ultra-fine grain steel has become the focus of research on high-strength materials. The methods of severe plastic deformation (SPD) are an effective means of grain refinement [1,2,3,4], which have been widely used in the preparation of ultra-high strength materials. The SPD methods mainly include cold rolling [5,6], equal channel angular pressing (ECAP) [7,8,9], high-pressure torsion (HPT) straining [10,11], accumulative roll-bonding (ARB) [12,13,14], multidirectional forging (MDF) [15,16,17], etc. Valiev et al. [18,19] reported the preparation and application of bulk nanostructured materials from SDP. Astafurova et al. [20] prepared high-strength steel with nanosized grain subgrain structure by HPT deformation of a low-alloy steel. Mousavi Anijdan et al. [21] treated Fe-28.5Ni steel by the ARB process and obtained the high-strength steel with a mean grain size of a few hundred nanometers after a 6-cycle process. Wang et al. [22] prepared ultra-fine-grained low carbon steel (Fe–0.15 wt% C–0.52 wt% Mn) with ultra-high strength by performing 10 times equal channel angular pressing (ECAP) at room temperature. Generally, severe cold deformation can increase the strength and hardness of the material while obviously reducing plasticity and toughness [18,19,20,21,22].

Martensitic transformation is another effective method to refine the grains of the materials, and so the strength level of the material can be greatly improved when the martensite structure is obtained [23,24,25,26]. The phase transformation strengthening of low carbon martensitic steel is mainly used to form fine lath martensite structure during quenching [27], and introduces high dislocation density [28,29] which can obtain high strength while retaining a certain plasticity to facilitate subsequent rolling deformation. An IF steel with two different initial structural states, namely ferrite and lath martensite, was cold rolled. When the cold rolling deformation reached to 80%, the UTS of the IF steel sheet with the initial ferrite state (grain size 150 μm) was about 616 MPa, while the UTS of the IF steel sheet with the initial lath martensite state reached to about 770 MPa [30]. Li et al. [31] performed cold rolling deformation on an interstitial free (IF) steel with coarse grain ferrite (120 μm). It was then found that the strength increased with increasing reduction, and the UTS was 530 MPa when the cold rolled reduction reached to 80%. These findings indicate that an IF steel with initial lath martensite can acquire higher strength than the IF steel with the initial ferrite state after the same plastic deformation. Astafurova et al. [20] prepared high-strength steel by HPT deformation of a low-alloy steel with two different initial structural states (ferritic-pearlitic state and martensitic state). They found that HPT deformation leads to a considerable increase in the microhardness compared to its microhardness in the initial state. Additionally, the hardness of the steel with an initial martensitic state was obviously higher than that of the steel with an initial ferritic-pearlitic state after the same HPT deformation.

At present, there are few studies on the preparation of ultra-high strength steel by small plastic deformation. The combination of martensitic transformation and plastic deformation can further enhance the grain refinement process while still maintaining good plasticity, which will be an effective way to prepare a high-strength low-carbon steel with higher strength grades. The ultra-high strength low-carbon steel will be obtained after plastic deformation at low strain when the initial structure of the steel is martensite.

In the present study, a plain AISI 1010 low-carbon steel is quenched to obtain a martensitic structure. Then the martensite lath structure was refined by plastic deformation at low strain, followed by an aging treatment to prepare 1600 MPa grade steel. The microstructural evolution and mechanical properties of the low-carbon steel are further examined and analyzed during the cold rolling deformation and subsequent aging treatment. This will provide the theoretical basis and experimental support for the preparation of ultra-high strength low carbon steel sheet.

## 2. Materials and Methods

**Figure 1:**
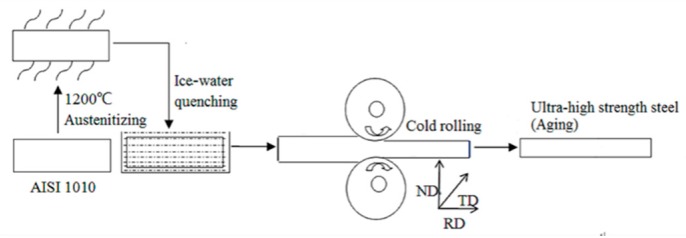
Preparation process of ultra-high strength low carbon steel.

A commercial plain low-carbon steel, namely AISI 1010 (0.12C-0.3Mn-0.15Cr-0.2Si, wt%), was used in this work. The sheets were 3 mm thick, 20 mm wide, and 50 mm long. The sheets were austenitized at 1200 °C for five min, followed by water quenching (at about 0 °C) to obtain the martensite structure. The sheets with martensitic microstructure were cold rolled to a reduction of 10% and 30% in thickness. The preparation process of the high-strength martensitic steel is depicted in Figure 1. The as-transformed specimens and the cold rolled specimens were subsequently aged at 120 °C.

**Figure 2:**
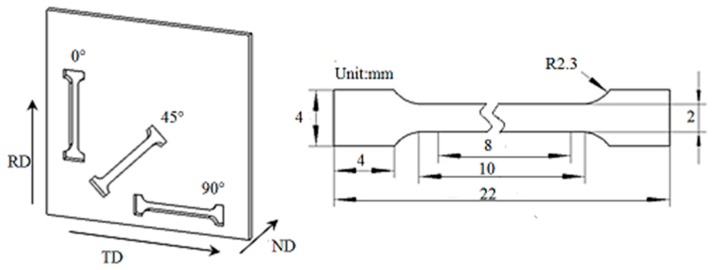
Sampling positions and dimensions of the tensile specimens.

The microstructure of each specimen was examined by various techniques. The structures were characterized by optical microscopy (OM), with an Axiovert-200MAT (Carl Zeiss Microscopy GmbH, Jena, Germany) and scanning electron microscopy (SEM) using a Hitachi S-3400N scanning electron microscope (Hitachi Corp., Tokyo, Japan) that the accelerating voltage was 20 KV. All specimens were observed on the rolling direction (RD)-normal direction (ND) plane of the sheets. Thin foils for TEM observation were cut into 6 mm long and 0.4 mm thickness by wire cutting equipment, mechanically thinned to 30 μm thickness, and electropolished by a twin-jet polisher with 10% perchloric acid solution at 30 V. TEM observations were performed with a Talos f200x (Thermo scientific TM, Hillsboro, OR, USA) transmission electron microscope with an acceleration voltage of 200 KV. The dislocation density of the material was analyzed by X-ray diffraction (XRD) on a D/max-2500PC X-ray diffractometer (Rigaku Corp, Tokyo, Japan), with Cu-K*α* (*λ* = 0.15406 nm) radiation at a scan rate of 2°/min, and operation voltage and current of 40 kV and 100 mA, respectively. Tensile specimens at different angles 0°, 45°, and 90° to the RD were selected from the steel sheets. The sampling positions and dimensions of the tensile specimens are shown in Figure 2. The tensile tests were conducted on a table Inspekt 100 kN universal material testing machine (Hegewald & Peschke MPT GmbH, Nossen, Germany), and the hardness was measured on an FM-700 FM-ARS microhardness tester (Future-Tech Corp., Kawasaki City, Kanagawa, Japan). The impact toughness test was carried out on Zwick RKP450 pendulum impact tester (Zwick GmbH & Co., Ulm, Germany) at room temperature. The impact specimens (cross-sectional dimension of about 10 mm thickness, 10 mm width, and 50 mm length) and Charpy U-notch impact specimens were assembled in multiple layers of thin plates of equal thickness. The approximate impact toughness of the sheets was obtained from the actual cross-sectional area calculated according to the actual thickness of the experimental sample. 

## 3. Results

### 3.1. Microstructure after Cold Rolling

**Figure 3:**
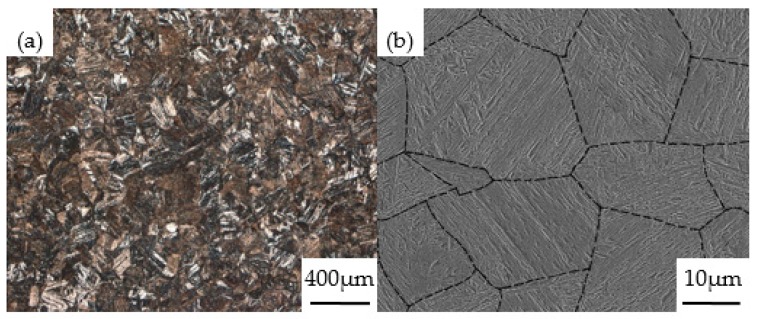
Optical microscopy (OM) image (**a**) and scanning electron microscopy (SEM) image (**b**) showing the microstructures of the as-quenched martensite in the experimental steel.

The quenched martensite of AISI 1010 steel is shown in Figure 3, revealing the typical lath martensite structure in the original austenite grains of the low-carbon steel. The parallel direction of the laths shows various angles with respect to the axes of the sample. The lath bundles are randomly distributed in multiple orientations and there is no specific angle with the subsequent deformation direction. 

**Figure 4:**
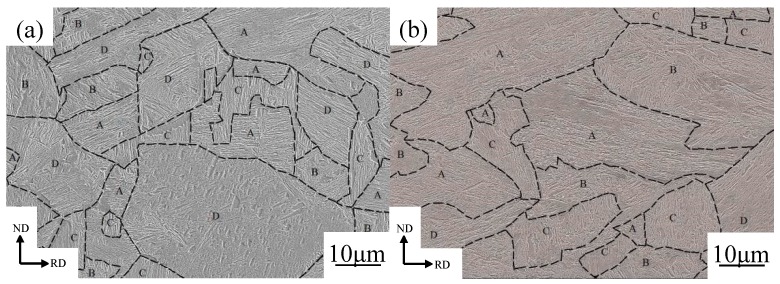
SEM images showing the microstructures of the experimental steel cold-rolled to a reduction of 10% (**a**) and 30% (**b**). The initial microstructure was martensite. Alphabetic characters denote which type of microstructure is exhibited. A, lamellar dislocation cell (LDC); B, irregularly bent lath (IBL); C, kinked lath (KL); D, lath martensite (M)).

The structure of lath martensite has been widely studied and reported in the literature. There is a three-level hierarchy in this morphology: (I) lath, a single crystal of martensite including high density lattice defects; (II) block, aggregation of laths with the same crystallographic orientation (variant); and (III) packet, aggregation of the blocks with the same habit plane [32,33,34,35]. The SEM images of the microstructure of the 10 and 30% cold rolled specimens are shown in Figure 4a,b, respectively. According to Ueji et al. [32], the structure of cold rolled martensite is divided into three kinds of microstructures, designated by the alphabetical characters (A, B, and C) and defined as follows:

A Very fine lamellar structure mainly elongated parallel to the RD.
B Irregularly bent lamellar structure.
C Lump of martensite laths with shear bands that is parallel to the ND.

The symbols of A, B, C, and D denote the lamellar dislocation cell (LDC), irregularly bent lath (IBL), kinked lath (KL) structures and lath martensite structures (M), in Figure 4, respectively. The broken lines in Figure 4 indicate the boundaries between the different structures with the A, B, C, and D microstructures. In the SEM image in Figure 4a reveals that the microstructures in the 10% cold-rolled specimen of low carbon martensitic steel is mainly M structure. The deformed microstructure is disordered and randomly distributed in different orientations and is at no specific angle with respect to the RD direction. The IBL and LDC structures were easily categorized by their characteristic morphology. However, the LDC and martensite structures both show similar lamellar structures, so they are sometimes difficult to distinguish. When the lamellar structure is elongated in a direction within 20° from the RD, it is considered as an LDC structure, otherwise the structure was categorized as a martensite structure (M) [33]. According to the images shown in Figure 4, all three kinds of deformed structures (LDC, IBL, and KL) occur in the 30% cold rolled specimens, and a fairly large amount of martensite structure still remains. The area occupied by the LDC, IBL, and KL structures increases at the same time, and the LDC structure occupies the largest area. The martensite lath also begins to be elongated along the RD.

**Figure 5:**
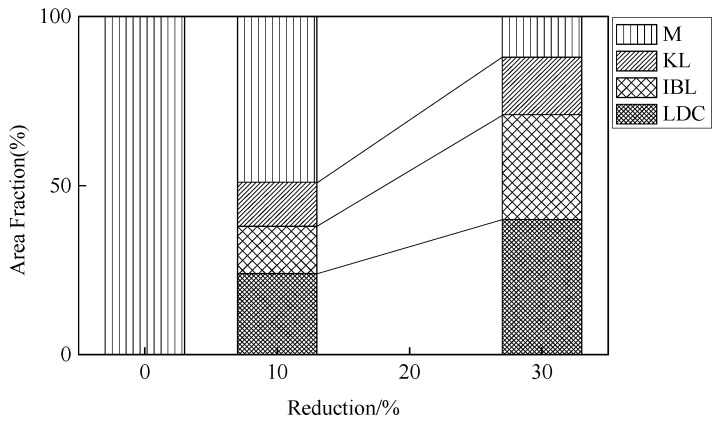
Fraction of the areas showing lamellar dislocation cells (LDCs), irregularly bent lath (IBL), kinked lath (KL), and lath martensite (M) in the sheets cold rolled to a reduction of 10% and 30%. The initial microstructure was martensite.

The as-quenched martensitic steel was cold rolled to a reduction of 10% and 30%, and the fractions of M, LDC, IBL, and KL of low-carbon martensitic steel under different rolling reductions are as shown in Figure 5. The fractions of the M, LDC, IBL, and KL structures in the 10% cold rolled sheet were 49%, 24%, 14% and 13%, respectively. The area fraction of the LDC, IBL, and KL increased with increasing the reduction, however, the area fraction of the M decreased. When the cold rolled reduction reaches to 30%, the area fraction of M decreases to 12%, but the area fraction of LDC, IBL, and KL increases to 40%, 31% and 17%, respectively. In other words, the martensite lath rotates with increasing cold rolled reduction. The lath is continuously elongated along the RD and gradually tends to become parallel to the RD. The martensitic microstructure is effective to obtain ultrafine grains, and the blocks and packets of martensite have angular and rugged shape [36]. Such high density of high-angle boundaries and complicated shape of blocks and packets would lead to inhomogeneous deformation (grain subdivision) during plastic deformation, which can result in an ultra-fine deformation microstructure with large local misorientations. After cold rolling deformation at low strains (Figure 4a,b), the martensitic structure mostly remains the same. 

### 3.2. Mechanical Properties after Cold Rolling

**Figure 6:**
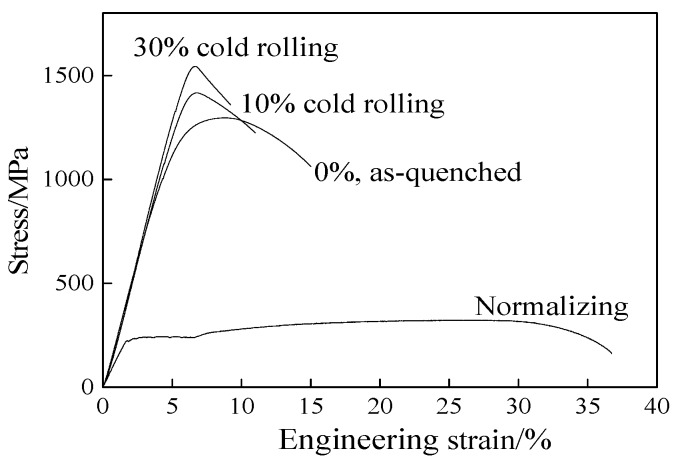
Tensile curves of the experimental steels at different states (quenched and cold rolled states).

The stress-strain curves of the as-quenched specimen and cold rolled specimens (10% and 30% reduction) are shown in Figure 6. The stress-strain curve of the normalized specimen is also shown in order to compare with the as-quenched specimen. The yield strength, tensile strength, and elongation of the experimental steel after normalizing (85 °C held for 10 min, air cooled) are 233 MPa, 320 MPa, and 32.2%, respectively. The results presented in Figure 6 reveal that cold deformation significantly enhances the strength and decreases the plasticity compared with the quenched specimen. The strength of the martensitic steel gradually increases, while the plasticity gradually decreases with increasing reduction. When the deformation reaches to 30%, the tensile strength of the low-carbon martensitic steel reaches to about 1600 MPa. 

**Figure 7:**
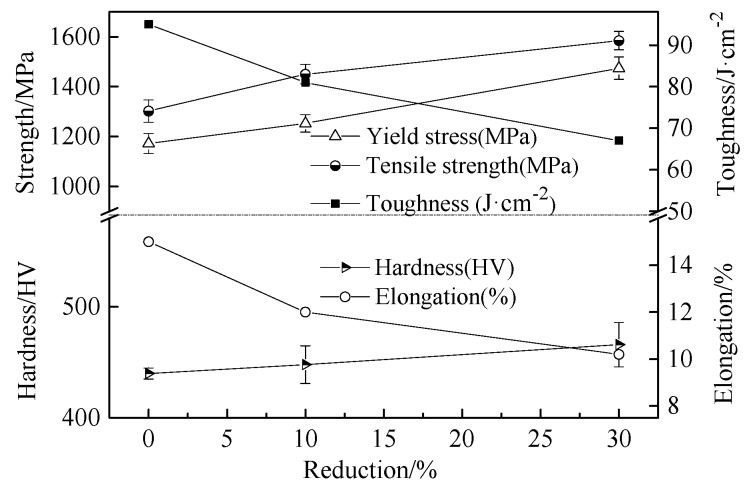
Mechanical properties of high-strength steel.

The yield strength, tensile strength, hardness, elongation, and impact toughness of the experimental steel after quenching and cold rolling are shown in Figure 7. After quenching, the strength and hardness markedly increased, while the elongation decreased compared with the normalized specimen. The hardness and tensile strength of the initial martensite were 440 HV and 1302 MPa, respectively.

After a cold rolled reduction of 30%, the hardness and tensile strength increase to 466 HV and 1585 MPa, respectively. The elongation and impact toughness of the quenched martensite were 15% and 95 J/cm^2^, respectively. With the increase of the cold rolled reduction to 30%, the elongation and impact toughness were reduced to 10.2% and 67 J/cm^2^, respectively. During the deformation process, the laths are also sheared by the micro-shear band while migrating toward the RD, thereby destroying the initial lath boundaries and causing a large misorientation within the martensite structure [30,32]. Then, the microstructure is further refined and the crystal defect density is gradually increased. As a result, the tensile strength and hardness of the experimental steel increase with the increasing cold rolled reduction, and the elongation tends to decrease. 

**Figure 8:**
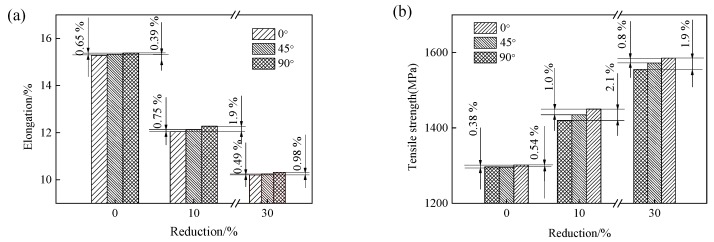
Elongation (**a**) and tensile strength (**b**) of low-carbon martensitic steel in the directions at angles 0°, 45°, and 90° with respect to the RD.

The tensile specimens were also selected in the direction of 0°, 45°, and 90° with respect to the RD of the experimental steel. The differences in the elongation and tensile strength of the experimental steel when the selected specimens were in different directions to the RD are shown in Figure 8. The strength along the 0° RD is little higher than that along the 45° and 90° RD, whereas the change in elongation is the opposite. 

The mechanical properties (elongation and strength) of the undeformed martensite steel at the different angles are almost the same, and the differences between them are very small (less than 0.7%). There is no obvious orientation concentration in the initial martensitic steel (Figure 3), and the martensite lath is randomly distributed in all directions. The deviations for the elongation and strength between 0° and 45° (or 0° and 90°) slightly increase after cold rolling. The martensite lath bundle underwent extension, bending, or torsion after cold rolling (Figure 4), which resulted in anisotropy of the structure in different orientations. 

When the cold rolled reduction reaches to 10%, the deviation of the elongation between 0° and 45° (or 0° and 90°) is 0.75% (or 1.9%), and the deviation of the strength between 0° and 45° (or 0° and 90°) is 1.0% (or 2.1%). When the cold rolled reduction reaches to 30%, the deviation of the elongation between 0° and 45° (or 0° and 90°) is 0.49% (or 0.98%), and the deviation of the strength between 0° and 45° (or 0° and 90°) is 0.8% (or 1.9%). These results reveal that the anisotropy of material properties is not obvious for the martensitic steel and the cold rolled martensitic steel at low strain (below 30% reduction). 

**Figure 9:**
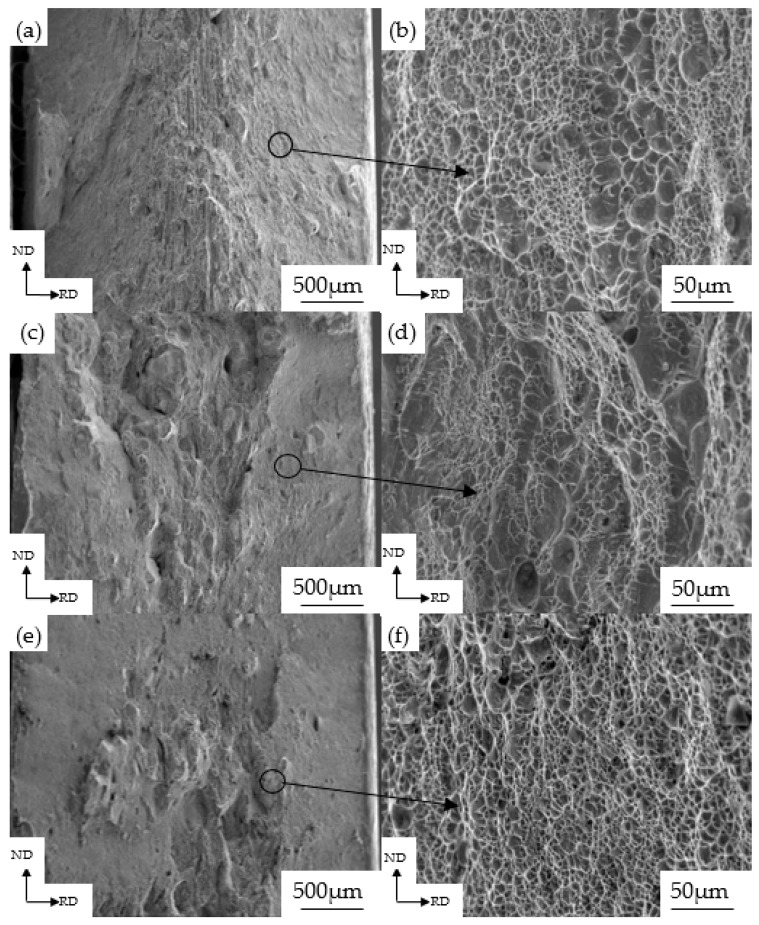
Tensile fracture morphology of the experimental steel quenched (**a**,**b**) and cold rolled to a reduction of 10% (**c**,**d**) or 30% (**e**,**f**).

The fracture morphology of the tensile specimens for the experimental steel is shown in Figure 9. Specifically, the fracture morphology of the as-quenched martensitic steel is shown in Figure 9a, which reveals that the fracture mode is mainly based on a ductile fracture, with fibers that are parallel to the initial fracture surface and arranged in rows. In the microscopic image of the fracture (Figure 9b), there are obvious equiaxed dimples, a large number of which are aggregated. The matching dimples are elongated in the same direction. Images of the fracture morphology of a 10% cold rolled specimen are shown in Figure 9c,d. Tearing edges appear at the heart of the fracture and the shear lip is mainly distributed around the fracture with a large number of voids. The dimple size and depth are gradually reduced compared with the as-quenched martensitic steel. When the reduction reaches 30%, there is a distinct tear-like structure in the core of the fracture displayed in Figure 9e. Compared with the 0% and 10% cold rolled specimens, the ductile fracture zone becomes very small and the material fracture mode is a mixed fracture mode. In Figure 9f, the dimple size and depth are significantly reduced.

### 3.3. Effects of Aging 

**Figure 10:**
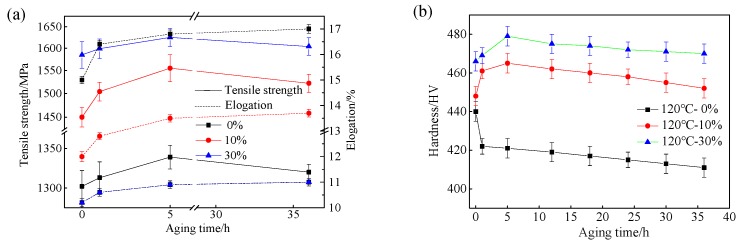
Tensile strength, elongation (**a**), and hardness (**b**) of the experimental steel after aging at 120 °C for various time periods.

The tensile strength, elongation and hardness of the specimens cold rolled and aging at 120 °C are shown in Figure 10a,b, respectively. The data in Figure 10a reveal that the tensile strength of the initial martensite and deformed martensite show an increasing trend at first, and then a decreasing trend with increasing aging time, and the tensile strength reaches a maximum value after aging for 5 h. The tensile strength of the as-quenched martensitic steel and the 30% cold rolled martensitic steel increases to 1339 and 1625 MPa, respectively, after aging treatment for 5 h. When the aging time reaches to 36 h, the tensile strength of the as-quenched martensitic steel and 30% cold rolled martensitic steel is 1320 and 1604 MPa, respectively. The elongation of the initial martensite and deformed martensite also increases with increasing aging time. When the aging treatment time reaches to 36 h, the elongation of the as-quenched martensitic steel increases from 15% to 17%, and the elongation of the 30% cold rolled specimen increases from 10.2% to 11%. The results in Figure 10b reveal that there are differences in the changes of the hardness with the aging time between the as-quenched and deformed martensite steel specimens. The hardness of the as-quenched specimen decreases gradually, decreasing from 440 to 411 HV with the increase of the aging time to 36 h. However, the hardness of the cold rolled deformed martensitic steel increases at first, but then decreases with increasing aging time. In particular, the hardness of the 30% cold rolled specimens increased from 466 to 479 HV after aging time for 5 h, but decreased gradually with increasing aging time. When the aging time reaches to 36 h, the hardness becomes 470 HV. 

**Figure 11:**
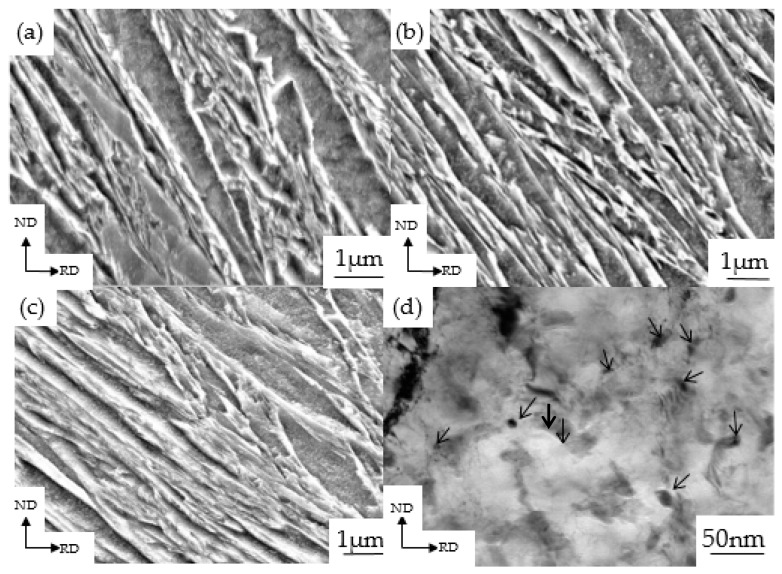
The SEM microstructures (**a**–**c**) and TEM microstructure (**d**) of the cold rolled sheets tempered at 120 °C for various time periods, (**a**) quenched specimens with subsequent aging for 5 h, (**b**) quenched specimens with subsequent aging for 36 h, (**c**) and (**d**) 30% cold rolled specimens with subsequent aging for 36 h (Arrows point to the precipitated nano-carbides).

The SEM images showing the microstructure of the as-quenched specimens and the 30% cold rolled specimens, and then aging at 120 °C for various time periods are displayed in Figure 11a–c. Specifically, Figure 11a,b show that after the aging treatment, the microstructures are not so different from the as-quenched one. The microstructure still exhibits the typical tempered lath martensite structures composed of recovered lath martensite. After aging at 120 °C for 36 h, a few fine carbides were found. The segregation and redistribution of carbon atoms take place into lattice defects such as dislocations, lath boundaries, and prior grain boundaries. In addition, the transitional epsilon-carbides also formed in this aging stage [37,38,39]. The locations of the precipitation of fine carbides are various throughout the deformed lath martensite and are worth studying further in the future. As shown in Figure 11c, when the reductions reached to 30%, the martensite phase interface becomes blurred and the substructure becomes coarser that the aging time was 36 h. Figure 11d showed a TEM image of a 30% cold rolled specimen after aging at 120 °C for 36 h, which can be clearly seen that the high-density dislocations still exist between the martensite laths and the lath martensite boundary becomes blurred. Carbides precipitated which showing a diffuse distribution. The carbide particles are small with size of 10–20 nm.

## 4. Discussion

The X-ray diffraction measurement results were used to analyze the samples under different conditions, and the spectra of the (110), (200), (211), and (220) crystal plane with relatively strong diffraction intensity were extracted. Jade software solves the full width at half maximum (FWHM) values of each peak adds the FWHM of the four crystal faces of the sample, and introduces the empirical Formula (1) [40] to calculate the dislocation density curve of different samples.

(1) $$\rho =\frac{\beta^{2}}{2\ln2\pi b^{2}}$$

where *ρ* is the dislocation density, *β* is the full width at half maximum of each peak, *b* is the Berk vector, where *b* = 0.247 nm [41].

**Figure 12:**
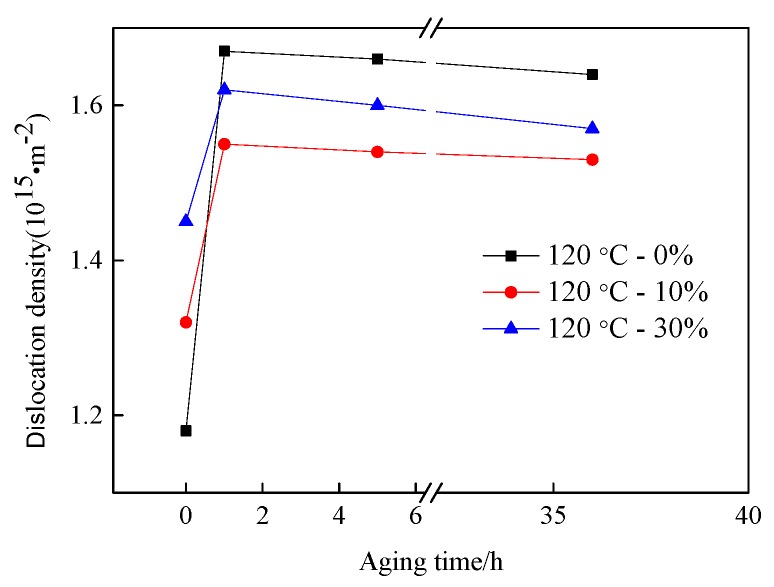
Dislocation density of the as-quenched and deformed martensitic steel with subsequent aging at 120 °C for various time periods.

The variation of the dislocation density for the as-quenched and deformed low-carbon martensitic steel as a function of the tempering time at 120 °C is shown in Figure 12. The dislocation density of the as-quenched and deformed low carbon martensitic steels showed a tendency to increase at first and then began to decrease after low temperature tempering for 1 h. Without aging treatment, the dislocation density of low-carbon martensitic steel increased from 1.18 × 10^15^ m^−2^ to 1.45 × 10^15^ m^−2^ with increasing cold rolled reduction. However, the dislocation density of the undeformed low-carbon martensitic steel was higher than that of the cold rolled specimen after aging treatment. When the aging time reached to 36 h, the dislocation density of the undeformed and deformed low-carbon martensitic steel were 1.64 × 10^15^ m^−2^ and 1.57 × 10^15^ m^−2^, respectively. The dislocation density of the cold rolled specimens continued to increase with the increase of the amount of cold rolled deformation after aging treatment. For the specimens with cold rolling of 10% and 30%, the dislocation density was 1.55 × 10^15^ m^−2^ and 1.62 × 10^15^ m^−2^ after aging treatment for 1 h, respectively. When the aging time increased to 36 h, the dislocation density was 1.53 × 10^15^ m^−2^ and 1.57 × 10^15^ m^−2^, respectively.

Since the carbon content of the experimental steel is low, when low-carbon martensite is tempered at 120 °C for 1 h, carbides will not precipitate after a short period of aging. Only carbon atoms will be segregated near the dislocations [42] and the Cottrell atmosphere will be formed. Speich [43] used calculations to find that in the case of steels containing less than 0.2 wt% C, almost 90% of the carbon segregates to dislocations and lath boundaries during quenching. When tempered at lower temperatures for a shorter period of time, these martensites cause additional segregation but no carbide precipitation. Due to the segregation of carbon atoms, result in an increase in dislocation density and tensile strength of martensitic steel after tempering for 1 h. When the tempering time exceeds 1 h, the recovery of the partial lath martensite occurred, and a small amount of supersaturated carbon precipitated from the martensite laths that can form *ε*-carbide and pinning movable dislocations [44,45,46]. At this time, the dislocation density began to decrease, but the tensile strength continued to increase. In other words, the phenomenon of “secondary hardening” occurred. After the aging time exceeded 5 h, as the aging time continued to increase, the martensite microstructure phase interface became blurred and the martensite lath bundles began to decompose. Additionally, the carbides precipitated between the martensite lath bundles began to undergo rearrangement and annihilation, which caused the martensite laths to coarsen. As a result, the dislocation density continued to decrease, and the tensile strength began to decrease. Caron and Krauss [47] pointed out that when fine carbides in the martensite laths were precipitated, the recovery was suppressed, causing the lath bundles to be effectively pinned and moved, thereby decreasing the dislocation density and resulted in the occurrence of the coarsen phenomenon. After aging for 1–36 h, the carbides gradually precipitated and the martensite laths gradually coarsened, which is the same as the conclusion obtained in reference [47]. After the aging treatment, the deformed martensite lath was coarsened (from Figure 11) which suppressed the recovery, so that the dislocation density of the undeformed martensitic steel was higher than the dislocation density of the cold rolled deformed martensitic steel.

The hardness and tensile strength for the deformed low-carbon martensite steel followed by aging were increased during the early aging time period, and when the aging time more than 5 h, the hardness (tensile strength) is reduced from 479 HV (1625 MPa) to 470 HV (1604 MPa), respectively, at 30% deformation. The variation of the tensile strength for the as-quenched martensite specimen is the same as that of the deformed one, but the hardness always decreased from 440 HV to 411 HV (Figure 10). The variation of the tensile strength and the hardness for the quenched martensite steel is different, indicating that a clear correspondence does not exist between the hardness and strength when the quenched steel was aging at low temperature. When the aging time reaches 36 h, the nano-carbide underwent a slight precipitation (Figure 11d). A large number of dislocations have been introduced during the martensitic transformation, and the structure of the martensite has been changed by cold deformation, which leads to an increase in the deformation dislocations. The diffusion channels of C atoms increase, and the diffusion activation energy of C atoms decreases, which may lead to the precipitation of nano-carbides in martensitic steel during low-temperature aging treatment. After martensitic transformation and cold deformation treatment followed by the low temperature aging treatment, the precipitation behavior of nano-carbides is needed to further research and discussion. At the same time, the dislocations migrated to form a dislocation interface in the recovery process that resulted in interface enhancement. The arrangement of the dislocation interface in the tensile process increased the strength of the lath martensitic steel, but the effect on the hardness was relatively small. Saeglitz and Krauss [48,49] have examined low temperature aging by varying time and temperature, and pointed out that for the low-carbon martensitic steels subjected to low-temperature aging treatment, the high hardness and strength are very much dependent on the density of the transition carbides and dislocations built into the low-temperature aging substructure of the tempered martensite crystals, and these carbon-dependent densities control the strain hardening that leads to high hardness and ultimate tensile strengths. Cahn et al. [50] pointed out that in brass, copper, or nickel with low stacking fault energy, there is no drop-in hardness during recovery, the recovery being accomplished by dislocation rearrangement, in contrast to aluminum and iron, where stacking fault energy is high and substantial decreases in hardness during recovery are accomplished apparently by reductions in dislocation density. The variation of the hardness and strength after aging treatment remains to be further studied. Huang et al. and Tsuji et al. [51,52] comparatively studied the cold rolled deformation of nanostructured pure aluminum and IF steel. After aging treatment, the stress of pure aluminum and IF steel increases while the dislocation density decreases, and this indicates that the presence of a certain amount of interior dislocations in the nanostructures produces softening rather than hardening as is observed in conventional coarse grained materials. This correlation between dislocation source density and intensity is usually observed in nanoscale metals through experiments [53] and through atomic models [54]. The availability of dislocation sources or the lack of such sources may therefore significantly affect the yield stress. The results of this study indicate that dislocations can cause softening and will have important applications in the study of new nanomaterials. The typical age hardening and over-aging softening behavior were observed through experiments. Due to the small content of experimental steel alloy elements used in this study, the changes of the mechanical properties are mainly related to the changes of dislocation motion and dislocation density. The softening phenomenon in the experiments of this paper is basically consistent with the experimental conclusions obtained in the literature.

## 5. Conclusions

The AISI 1010 low-carbon steel was quenched with ice water to obtain the lath martensite structure, followed by cold rolling at low strains (<30%) and aging at low temperature (120 °C). The mechanical behavior of the deformed martensitic structure was studied. The microstructural evolution of the martensite was examined. The major results are summarized as follows:

(1) The deformed structures (LDC, IBL, and KL) were present in the 10% and 30% cold rolled specimens and fairly large amount of the martensite structure remained the same. The area occupied by the LDC, IBL and KL structure increased from 24, 14, and 13% to 40, 31, and 17% with increasing rolling reductions. The martensite lath began to be elongated along the RD and the martensite structure was gradually refined.
(2) As the reduction increased, the hardness and the tensile strength gradually increased, and the elongation decreased. When the reduction reached to 30%, the hardness and tensile strength increased to 466 HV and 1585 MPa, respectively. However, the elongation decreased to 10.2%. When the tensile specimens were selected at 0°, 45°, and 90° in the RD, the specimens exhibited different strength and elongation. The deviation was calculated to be within 3%. The anisotropy of the material properties was not obvious for the martensitic steel and the cold rolled martensitic steel at low strain (below 30% reduction).
(3) The strength of the initial martensite and deformed martensite showed an increasing trend at first, and then a decreasing trend with increasing aging time, the tensile strength reached to a maximum value after aging for 5 h. The tensile strength of the as-quenched martensitic steel and 30% cold rolled martensitic steel increased to 1339 and 1625 MPa after aging treatment for 5 h, respectively. The elongation of the initial martensite and deformed martensite increased with increasing aging time. When the aging treatment time reached to 36 h, the elongation of the as-quenched martensitic steel and 30% cold rolled specimen increased to 17 and 11%, respectively.

## References

[1] Dong H.S., Kim B.C., Park K.T., Choo W.Y. (2000). Microstructural changes in equal channel angular pressed low carbon steel by static annealing. Acta Mater..

[2] Kaibyshev O.A. (2001). Grain refinement in commercial alloys due to high plastic deformations and phase transformations. J Mater. Process. Technol..

[3] Morovvati M.R., Mollaei-Dariani B. (2018). The formability investigation of CNT-reinforced aluminum nano-composite sheets manufactured by accumulative roll bonding. Int. J. Adv. Manu. Technol..

[4] Sauvage X., Ganeev A., Ivanisenko Y., Enikeev N., Murashkin M., Valiev R.Z. (2012). Grain Boundary Segregation in UFG Alloys Processed by Severe Plastic Deformation. Adv. Eng. Mat..

[5] Ghassemali E., Kermanpur A., Najafizadeh A. (2010). Microstructural Evolution in a Low Carbon Steel During Cold Rolling and Subsequent Annealing. J. Nanosci. Nanotechnol..

[6] Lv Z., Jiang P., Wang Z., Zhang W., Sun S., Fu W. (2008). XRD analyses on dissolution behavior of cementite in eutectoid pearlitic steel during cold rolling. Mater. Lett..

[7] Fukuda Y., Oh-Ishi K., Horita Z., Langdon T.G. (2002). Processing of a low-carbon steel by equal-channel angular pressing. Acta Mater..

[8] He T., Xiong Y., Ren F., Guo Z., Volinsky A.A. (2012). Microstructure of ultra-fine-grained high carbon steel prepared by equal channel angular pressing. Mat. Sci. Eng. A-Struct..

[9] Xu J., Li J., Zhu X., Fan G., Shan D., Guo B. (2015). Microstructural Evolution at Micro/Meso-Scale in, an Ultrafine-Grained Pure Aluminum Processed by Equal-Channel Angular Pressing with Subsequent Annealing Treatment. Materials.

[10] Dobathin S.V., Shagalina S.V., Sleptsov O.I., Krasil’nikov N.A. (2006). Effect of the initial state of a low-carbon steel on nanostructure formation during high-pressure torsion at high strains and pressures. Russ. Metall..

[11] Song R., Pong D., Raabe D., Speera J.G., Matlocka D.K. (2006). Overview of processing, microsture and mechanical properties of ultrafine grained bcc steels. Mater. Sci. Eng. A-Struct..

[12] Kwan C., Wang Z. (2013). Cyclic Deformation of Ultra-Fine Grained Commercial Purity Aluminum Processed by Accumulative Roll-Bonding. Materials.

[13] Su L., Lu C., Tieu K., Deng G. (2013). Annealing Behavior of Accumulative Roll Bonding Processed Aluminum Composites. Steel Res. Int..

[14] Saito Y., Utsunomiya H., Tsuji N., Sakai T. (1999). Novel ultra-high straining process for bulk materials—development of the accumulative roll-bonding (ARB) process. Acta Mater..

[15] Kobayashi C., Sakai T., Belyakov A., Miura H. (2007). Ultrafine grain development in copper during multidirectional forging at 195 K. Philos. Mag. Lett..

[16] Wang B., Wang X., Li J. (2016). Formation and Microstructure of Ultrafine-Grained Titanium Processed by Multi-Directional Forging. J. Mater. Eng. Perform..

[17] Salishchev G., Zaripov R., Galeev R., Valiakhmetov O. (1999). Nanocrystalline structure formation during severe plastic deformation in metals and their deformation behaviour. Nanostruct. Mater..

[18] Valiev R.Z., Islamgaliev R.K., Alexandrov I.V. (2000). Bulk nanostructured materials from severe plastic deformation. Prog. Mater. Sci..

[19] Valiev R.Z., Ivanisenko Y.V., Rauch E.F., Baudelet B. (1996). Structure and deformaton behaviour of Armco iron subjected to severe plastic deformation. Acta Mater..

[20] Astafurova E.G., Dobatkin S.V., Naydenkin E.V., Shagalina S.V., Zakharova G.G., Ivanov Y.F. (2009). Structural and phase transformations in nanostructured 0.1% C-Mn-V-Ti steel during cold deformation by high pressure torsion and subsequent heating. Nanotechnol. Russ..

[21] Mousavi A.S.H., Jafarian H.R., Park N. (2018). The effect of severe plastic deformation and annealing conditions on mechanical properties and restoration phenomena in an ultrafine-grains Fe-28.5%Ni steel. Philos. Mag..

[22] Wang J.T., Xu C., Du Z.Z., Qu G.Z., Langdon T.G. (2005). Microstructure and properties of a low-carbon steel processed by equal-channel angular pressing. Mat. Sci. Eng. A-Struct..

[23] Senuma T. (2001). Advances in Physical Metallurgy and Processing of Steels. Physical Metallurgy of Modern High Strength Steel Sheets. ISIJ Int..

[24] Morito S., Yoshida H., Maki T., Huang X. (2006). Effect of block size on the strength of lath martensite in low carbon steels. Mater. Sci. Eng..

[25] Galindo-Nava E.I., Rivera-Díaz-del-Castillo P.E.J. (2015). A model for the microstructure behaviour and strength evolution in lath martensite. Acta Mater..

[26] Hutchinson B., Hagström J., Karlsson O., Lindell D., Tornberg M., Lindberg F., Thuvander M. (2011). Microstructures and hardness of as-quenched martensites (0.1–0.5%C). Acta Mater..

[27] Morito S., Nishikawa J., Maki T. (2003). Dislocation density within lath martensite in Fe-C and Fe-Ni alloy. ISIJ Int..

[28] Christien F., Telling M.T.F., Knight K.S. (2013). Neutron diffraction in situ monitoring of the dislocation density during martensitic transformation in a stainless steel. Scr. Mater..

[29] Krauss G. (2005). Steels: Processing, Structure, and Performance.

[30] Huang X., Morito S., Hansen N., Maki T. (2012). Ultrafine Structure and High Strength in Cold-Rolled Martensite. Metall. Mater. Trans..

[31] Li B.L., Godfrey A., Meng Q.C., Liu Q., Hansen N. (2004). Microstructural evolution of IF-steel during cold rolling. Acta Mater..

[32] Ueji R., Tsuji N., Minamino Y., Koizumi Y. (2002). Ultragrain refinement of plain low carbon steel by cold-rolling and annealing of martensite. Acta Mater..

[33] Ueji R., Tsuji N., Minamino Y., Koizumi Y. (2004). Effect of rolling reduction on ultrafine grained structure and mechanical properties of low-carbon steel thermomechanically processed from martensite starting structure. Sci. Technol. Adv. Mater..

[34] Maki T. (1980). The Morphology of Micro-structure Composed of Lath Martensites in Steels. Trans. ISIJ.

[35] Morito S., Tanaka H., Furuhara T., Maki T. Recovery and Recrystallization of Lath Martensite in an Ultra-low Carbon Steel. Proceedings of the 4th International Conference on Recrystallization and Related Phenomena (RECRYSTALLIZATION 99).

[36] Tsuji N., Ueji R., Minamino Y., Saito Y. (2002). A new and simple process to obtain nano-structured bulk low-carbon steel with superior mechanical property. Scr. Mater..

[37] Saha D.C., Biro E., Gerlich A.P., Zhou Y. (2016). Effects of tempering mode on the structural changes of martensite. Mater. Sci. Eng. A..

[38] Thomson R.C., Miller M.K. (1998). Carbide precipitation in martensite during the early stages of tempering Cr- and Mo-containing low alloy steels. Acta Mater..

[39] Jung M., Lee S.J., Lee Y.K. (2009). Microstructural and Dilatational Changes during Tempering and Tempering Kinetics in Martensitic Medium-Carbon Steel. Metal. Mater. Trans. A.

[40] Gay P., Hirsch P.B., Kelly A. (1953). The estimation of dislocation densities in metals from X-ray data. Acta Metal..

[41] Williamson G.K., Smallman R.E. (1956). Dislocation densities in some annealed and cold-worked metals from measurements on the X-ray debye-scherrer spectrum. Philos. Mag..

[42] Wilde J., Cerezo A., Smith G.D.W. (2000). Three-dimensional atomic-scale mapping of a cottrell atmosphere around a dislocation in iron. Scr. Mater..

[43] Speich G.R. (1969). Tempering of low-carbon martensite. Trans. TMS-AIME.

[44] Taylor K.A., Olson G.B., Cohen M., Sande J.B.V. (1989). Carbide precipitation during stage Ⅰ tempering of Fe-Ni-C martensites. Metall. Mater. Trans. A.

[45] Krauss G. (2017). Tempering of Lath Martensite in Low and Medium Carbon Steels. Assessment and Challenges. Steel Res. Int..

[46] Yang G.W., Yong Q.L., Sun X.J., Li Z. (2013). Effects of process on microstructure and mechanical properties of 1500 MPa grade low carbon Nb-Ti low alloy steel. Cailiao Rechuli Xuebao/Trans. Mater Heat Treat..

[47] Caron R.N., Krauss G. (1972). The tempering of Fe-C lath martensite. Metall. Trans..

[48] Saeglitz M., Krauss G. (1997). Deformation, fracture, and mechanical properties of low-temperature-tempered martensite in SAE 43xx steel. Metall. Mater. Trans. A.

[49] Krauss G. (2001). Deformation and fracture inmartensitic carbon steels tempered at low temperatures. Metall. Mater. Trans. B.

[50] Cahn R.W., Haasen P. (1983). Physical Metallurgy.

[51] Huang X., Kamikawa N., Tsuji N., Hansen N. (2008). Nanostructured Aluminum and IF Steel Produced by Rolling-a Comparative Study. ISIJ Int..

[52] Huang X., Hansen N., Tsuji N. (2006). Hardening by annealing and softening by deformation in nanostructured metals. Science.

[53] Greer J.R., Oliver W.C., Nix W.D. (2005). Size dependence of mechanical properties of gold at the micron scale in the absence of strain gradients. Acta Mater..

[54] Schiotz J., Jacobsen K.W. (2003). A maximum in the strength of nanocrystalline copper. Jacobsen Sci..

